# Cation Disorder in Ferroelectric Ba_4_M_2_Nb_10_O_30_ (M = Na, K, and Rb) Tetragonal Tungsten Bronzes

**DOI:** 10.1021/acs.inorgchem.2c02266

**Published:** 2022-09-22

**Authors:** Inger-Emma Nylund, Nora Statle Løndal, Julian Walker, Per Erik Vullum, Mari-Ann Einarsrud, Tor Grande

**Affiliations:** † Department of Materials Science and Engineering, NTNU Norwegian University of Science and Technology, 7491 Trondheim, Norway; ‡ Department of Physics, NTNU Norwegian University of Science and Technology, 7491 Trondheim, Norway; § SINTEF Industry, NO-7034 Trondheim, Norway

## Abstract

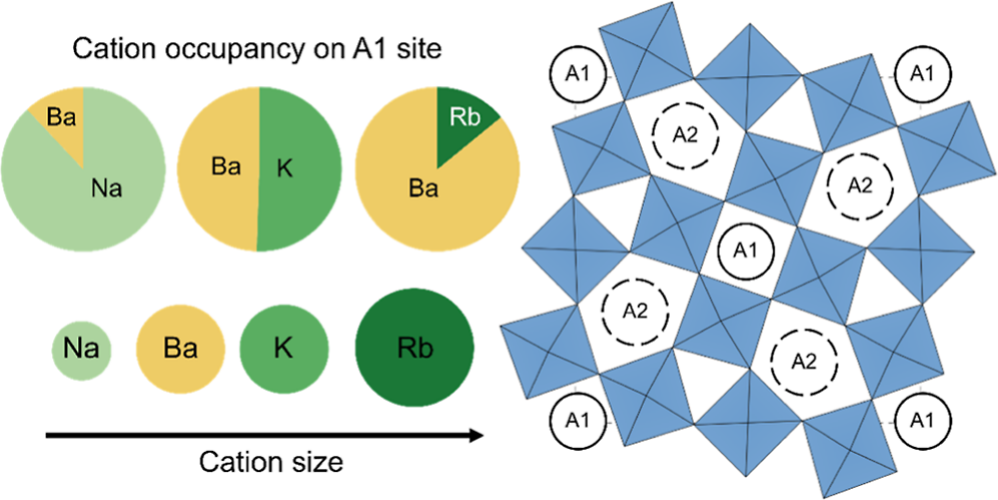

The crystal structure of tetragonal tungsten bronzes, with the general formula A1_2_A2_4_C_4_B1_2_B2_8_O_30_, is flexible both from a chemical and structural viewpoint, resulting in a multitude of compositions. The A1 and A2 lattice sites, with different coordination environments, are usually regarded to be occupied by two different cations such as in Ba_4_Na_2_Nb_10_O_30_ with Na^+^ and Ba^2+^ occupying the A1 and A2 sites, respectively. Here, we report on a systematic study of the lattice site occupancy on the A1 and A2 sites in the series Ba_4_M_2_Nb_10_O_30_ (M = Na, K, and Rb). The three compounds were synthesized by a two-step solid-state method. The site occupancy on the A1 and A2 sites were investigated by a combination of Rietveld refinement of X-ray diffraction patterns and scanning transmission electron microscopy with simultaneous energy-dispersive spectroscopy. The two methods demonstrated consistent site occupancy of the cations on the A1 and A2 sites, rationalized by the variation in the size of the alkali cations. The cation order–disorder phenomenology in the tungsten bronzes reported is discussed using a thermodynamic model of O’Neill and Navrotsky, originally developed for cation interchange in spinels.

## Abstract 2

In tetragonal tungsten bronzes, with general formula A1_2_A2_4_C_4_B1_2_B2_8_O_30_, the A1 and A2 lattice sites are usually regarded to be occupied by two different cations. Here, the site occupancy on the A1 and A2 sites in the tetragonal tungsten bronzes Ba_4_M_2_Nb_10_O_30_ (M = Na, K, and Rb) was investigated by a combination of Rietveld refinement of X-ray diffraction data and scanning transmission electron microscopy with simultaneous energy-dispersive spectroscopy, revealing cation intermixing rationalized by the relative size difference between the two cations.

## Introduction

Ferroelectric tetragonal tungsten bronzes (TTBs) are the second largest group of ferroelectric oxide materials after the perovskites.^1^ The TTB crystal structure resembles the perovskite structure as it consists of corner-sharing BO_6_ octahedra; however, the unit cell of the prototypical TTB is approximately 10 times larger than the unit cell of the perovskite. The BO_6_ octahedra in the TTB structure are connected such that four triangular, two square, and four pentagonal channels are present in each unit cell viewed along the *c*-axis. The square site has a coordination number (CN) of 12 and is denoted as A1, and the pentagonal site has CN = 15 and is denoted as A2. In a filled TTB, the triangular channels are empty, whereas the square and pentagonal channels are fully occupied, and the general formula can be written as A2_4_A1_2_B_10_O_30_.^2^ The different cation lattice sites open up for a rich chemistry of the TTBs, and a large family of ferroelectric materials has been reported, including relaxor ferroelectrics.^3^

The cations with the larger ionic radius are expected to occupy the larger lattice sites in the crystal structure, hence the largest A-cation will preferentially occupy the A2 site in the TTB structure. For example, in an early work on the TTB compound Ba_4_Na_2_Nb_10_O_30_ (BNN) it was explicitly stated that the larger Ba^2+^ cation occupies the A2 sites, whereas the smaller Na^+^ cation occupies the A1 sites.^4^ More recently, a Rietveld refinement of X-ray diffraction (XRD) data on BNN demonstrated that configurational cation disorder is present, such that there is significant intermixing of Ba and Na between the A1 and A2 sites, which was further supported by density functional theory (DFT) calculations.^5^ In the related compounds Ba_4_K_2_Nb_10_O_30_ (BKN) and Ba_4_Rb_2_Nb_10_O_30_ (BRN), pronounced cation disorder is anticipated due to the more similar size of the cations, but a high degree of ordering of these compounds was inferred by the  ratio of the unit cells.^6^ The energetics of cation disorder in TTBs has recently been investigated by DFT in combination with thermodynamic models for cation disorder.^7^ The thermodynamic model used was adapted by a model developed to describe the more known cation disorder in spinels (AB_2_O_4_).^8^

Cation disorder in TTBs has so far to a large degree been neglected, but it has recently been demonstrated that cation disorder takes place in TTBs and may also affect functional properties such as the Curie temperature.^9,10^ In this study, we report the first systematic study of cation disorder in TTBs by changing the cation sizes in the series of compounds BNN, BKN, and BRN. Powder XRD and scanning transmission electron microscopy with simultaneous energy-dispersive spectroscopy (STEM–EDS) were performed to evaluate the degree of cation disorder. We demonstrate that the observed degree of disorder on the A1 and A2 cation sites in these three compounds can be rationalized by the variation in the size of the alkali cations.

## Experimental Section

BMN (M = Na, K, or Rb) ceramics were synthesized using BaCO_3_ (99+ %, ThermoFisher), Nb_2_O_5_ (>99.99%), and either Na_2_CO_3_ (≥99.0%), K_2_CO_3_ (≥99.0%), or Rb_2_CO_3_ (99%), respectively, as precursors (Sigma-Aldrich). To avoid extensive loss of the volatile alkali cations during processing at high temperature, powders of NaNbO_3_, KNbO_3_, and RbNbO_3_ were first prepared by mixing the appropriate precursors in ethanol by ball milling. The precursor mixtures were dried, uniaxially pressed to pellets at approximately 30 MPa, and calcined first at 700 °C for 4 h before further calcination at 900 °C for 12 h. BaNb_2_O_6_ was prepared by a similar procedure with calcination at 1100 °C for 6 h. BNN was prepared by mixing NaNbO_3_ with BaNb_2_O_6_ in ethanol by ball milling, dried, pressed to pellets at approximately 70 MPa, and sintered at 1285 °C for 6 h with a heating rate of 200 °C/h. To avoid loss of alkali oxide during sintering, the pellets were covered by sacrificial powder of the same composition. The two other tungsten bronzes were prepared by the same procedure, with adjusted sintering parameters, BKN (1275 °C, 6 h, 300 °C/h) and BRN (1150 °C, 2 h, 200 °C/h).

Approximately 100 μm of surface was polished off the sintered pellets to remove possible secondary phases formed at the surface due to the known volatility of the alkali oxides. Powders were prepared by crushing and hand grinding in a carbide mortar. To prepare TEM specimens, the fine powders were dispersed in ethanol or isopropanol and dropped onto a Cu grid covered with holey amorphous carbon film.

Powder XRD patterns of BNN, BKN, and BRN were measured with a Bruker DaVinci1 diffractometer with Cu Kα radiation, and Rietveld refinement of the diffraction patterns were performed for all three powders. The BNN data were refined using a *Cmm*2^5^ structure model and all symmetry inequivalent ion positions were refined. For BKN and BRN, a *P*4*bm* structure^10,11^ was used in the refinement and symmetry inequivalent ion positions for Ba, M, and Nb were refined, whereas all the O-positions were not refined and kept constant to the initial value. The stoichiometrically constricted occupancies for Ba and M were refined, as described in Table S1 in Supporting Information. The atomic displacement parameters (*B*_eq_) used in the refinements were taken from the combined X-ray and neutron diffraction study by Olsen et al.^12^ for all three compounds and were kept constant throughout the refinements. A fundamental parameter model described the peak shape, and the background was described by a Chebyshev polynomial of minimal degree.

Simultaneous STEM and EDS (STEM–EDS) was performed using a coldFEG Jeol ARM200F aberration-corrected microscope equipped with a Centurio SDD EDS detector (nominal solid angle, 0.98 sr). The microscope was operated at 200 kV with a beam semi-convergence angle of 27.4 mrad. High-angle annular dark-field–STEM (HAADF–STEM) images were acquired with collection angles of 51–203 mrad, and the probe current was about 120 pA. HAADF–STEM images and spectral images were acquired simultaneously by scanning 256 × 256 pixels, with a step size of 0.3 Å. To avoid beam damage, the pixel scan time was 0.001 s, resulting in a frame time of only 1 min 6 s. For each specimen, however, multiple spectral images were acquired at a few different areas within approximately 20 nm and superimposed by SmartAlign.^13^ In SmartAlign, image alignment and scan distortion compensation were performed on the HAADF–STEM images and the corrections were transferred to the spectral images. The fast-scan direction differed by 5–10° compared to a major crystallographic axis to maximize the precision of the SmartAlign procedure. Due to the corrections performed by SmartAlign, the resulting spectral images are cropped compared to the originally acquired images. For all the three specimens, 60 spectral images were acquired over a time span of about 2 h. However, 12, 16, and 21 frames were discarded for BNN, BKN, and BRN, respectively, due to severe distortions appearing during scanning. Focus and astigmatism were adjusted between each frame, when needed, and the beam was blanked to avoid irradiating the sample while the data was saved (which took approximately 20 s for each frame). The spectral images were then analyzed using the HyperSpy v1.5.2^14^ and Atomap v0.1.4^15^ packages in Python. Quantification was done using the Cliff–Lorimer (CL)^16^ quantification procedure with *k*-factors taken from the EDS software in DigitalMicrograph. The Ba_Lα_, M_Kα_, Nb_Kα_, and O_Kα_ peaks were used for quantification. Spectra acquired at each A1 and A2 sublattice were summed up to ensure enough intensity for the quantification procedure. For all specimens, it was assumed that Ba and M made up 100% of the concentration at the A1 and A2 sites. Further details on the analysis are presented in the Supporting Information.

## Results

**Figure 1:**
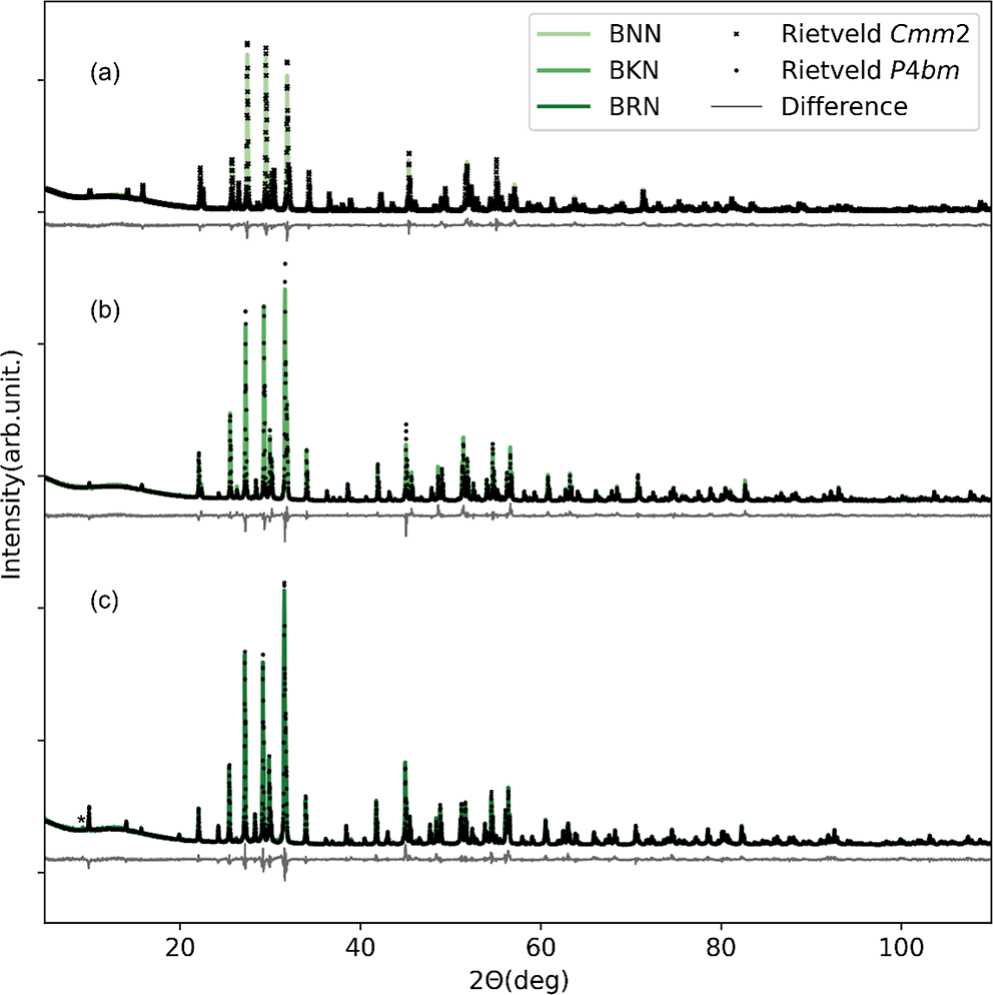
Rietveld refinement of powder XRD patterns of (a) BNN using the *Cmm*2 structure, and of (b) BKN and (c) BRN using the *P*4*bm* structure. In BRN, minor traces of the secondary phase Rb_4_Nb_6_O_17_·3H_2_O is present and marked with * at ∼ 9° 2θ.

XRD patterns of BNN, BKN, and BRN are shown in Figure 1 together with the Rietveld refinement of the diffraction data. The parameters determined by the Rietveld refinements are summarized in Tables S2–S4 in Supporting Information. BNN and BKN were phase pure, whereas minor traces of the secondary phase Rb_4_Nb_6_O_17_·3H_2_O was identified in BRN. The only visible reflection of this secondary phase is marked with an asterisk in Figure 1c. The refined occupancies for Ba and M on the A1 and A2 sites, respectively, for each of the three compounds are summarized in Table 1.

**Table 1:** Occupancy of Ba and the Respective Alkali Metal at the A1 and A2 Sites in BNN, BKN, and BRN Obtained by Rietveld Refinements and STEM–EDS Measurements

compound	site	atom	occupancy (XRD)	occupancy (STEM–EDS)
BNN	A2	Ba	0.9398(18)	0.8(2)
		Na	0.0602(18)	0.2(2)
	A1	Na	0.880(4)	0.6(2)
		Ba	0.120(4)	0.4(2)
BKN	A2	Ba	0.7525(19)	0.7(1)
		K	0.2475(19)	0.3(1)
	A1	K	0.505(4)	0.4(1)
		Ba	0.495(4)	0.6(1)
BRN	A2	Ba	0.570(4)	0.6(1)
		Rb	0.430(4)	0.4(1)
	A1	Rb	0.141(7)	0.2(1)
		Ba	0.859(7)	0.8(1)

The total EDS signal in the spectral images for the three compounds were summed up to one spectrum, respectively, which is shown in Figure S1 in Supporting Information. The total composition of the three compounds calculated from the CL quantification based on the summed spectra is presented in Table 2, together with the concentrations corresponding to the stoichiometric compounds. The calculated ratio between Ba and M is also presented in Table 2.

**Table 2:** Calculated Chemical Composition of BNN, BKN, and BRN from the CL Quantification of the Summed Spectra Acquired by STEM–EDS Together with Concentrations Corresponding to the Stoichiometric Compounds

compound	Ba [at %]	M [at %]	Nb [at %]	O [at %]	Ba/M
theoretical	8.7	4.4	21.7	65.2	2
BNN (M = Na)	8.0	2.8	21.1	68.1	2.9
BKN (M = K)	11.0	4.1	27.7	57.2	2.7
BRN (M = Rb)	14.6	5.6	38.9	40.8	2.6

**Figure 2:**
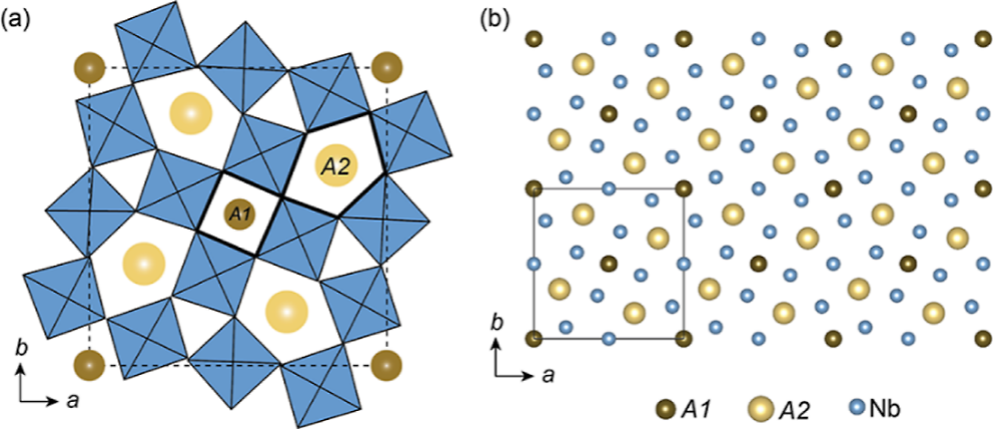
Schematic of the TTB structure. (a) One unit cell of the TTB structure where the square A1 and pentagonal A2 sites are indicated. (b) Six unit cells of the TTB structure viewed along the *c*-axis where the A1, A2, and Nb sites are shown to demonstrate how the structure appears in the HAADF–STEM image. The figure in (b) is made using Vesta.^17^

A schematic overview of the TTB structure is displayed in Figure 2. One unit cell of the prototype TTB is shown in Figure 2a, where the A1 and A2 sites are indicated. Figure 2b provides an overview of six unit cells viewed along the *c*-axis, where the thin black square shows the size of one unit cell. Only the cations are shown, as only the cations result in a sufficient contrast in the HAADF–STEM images.

**Figure 3:**
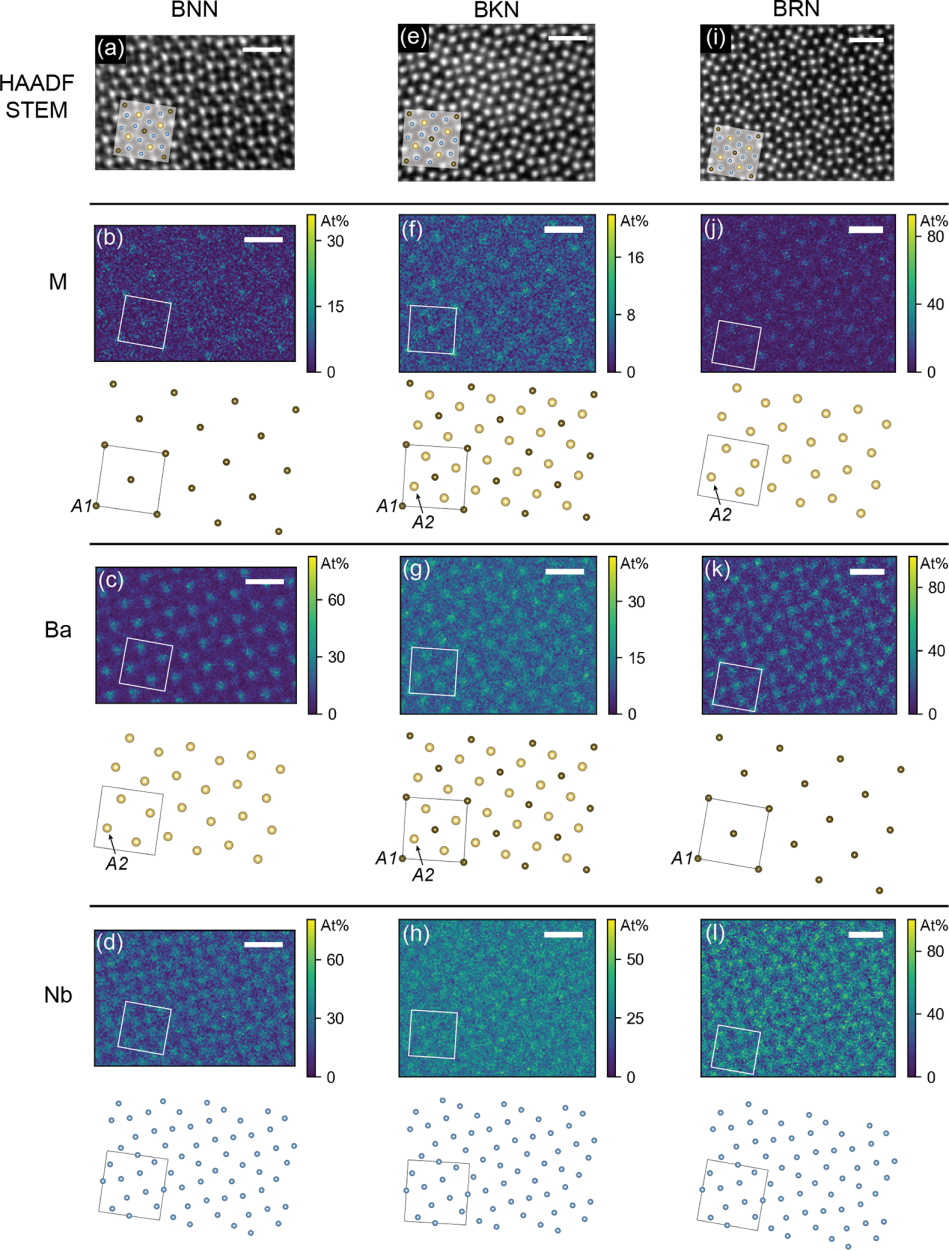
HAADF–STEM images and compositional maps for BNN (a–d), BKN (e–h), and BRN (i–l), viewed along the *c*-axis. An atomic sublattice is shown below each map indicating the main site for each element. In BNN, (b) Na occupies mainly the A1 site and (c) Ba mainly occupies the A2 site. In RBN, (j) Rb shows the highest concentration at the A2 site, whereas the concentration of (k) Ba is highest at the A1 site. BKN shows an intermediate intermixing compared to BNN and BRN. The distance between the Nb columns is much shorter than the distance between the A1 and A2 columns, respectively, thus the individual atomic columns are not as distinct in the Nb maps. The white square in each elemental map corresponds to the black square in the atomic sublattice below. Scale bars = 1 nm.

To get an overview of the degree of cation intermixing for the different compounds, pixel-by-pixel CL quantification was performed on the spectral images as presented in Figure 3. Each column in the figure shows the data from each compound, respectively, starting with BNN in the left column, BKN in the middle, and lastly BRN in the right column. The first row (Figure 3a,e,i) shows the HAADF–STEM images processed using SmartAlign, the second row (Figure 3b,f,j) displays the calculated at % of M = Na, K, and Rb, respectively, the third row (Figure 3c,g,k) shows the Ba concentration for each compound, and finally the fourth row (Figure 3d,h,l) gives the Nb concentration.

Various sublattices of the crystal structure displayed in Figure 2b are repeated in Figure 3 to illustrate the lattice sites which have the highest concentration of the different elements. A clear difference can be seen between BNN and BRN. Looking first at the Ba concentration in the two materials, Figure 3c demonstrates that Ba is mainly found on the A2 site in BNN, whereas the concentration of Ba is higher on the A1 site in BRN as seen in Figure 3k. Figure 3k also demonstrates that there is presence of a lower concentration of Ba at the A2 sites in BRN. Regarding the Na concentration in BNN (Figure 3b) and Rb concentration in BRN (Figure 3j), the concentration maps evidence an increased concentration of Na at the A1 site, whereas Rb is mainly found on the A2 site. Ba and K can be seen to occupy both the A1 and A2 sites in BKN, although it appears as K can be found at a higher concentration on the A1 site (Figure 3f), whereas Ba is found at a higher concentration at the A2 site (Figure 3g). The Nb concentration maps are shown for all three compounds for completion. Because the distance between each Nb column is smaller than the distances between the Ba columns and the M columns, single columns are not as easily distinguished for Nb as for the other elements.

**Figure 4:**
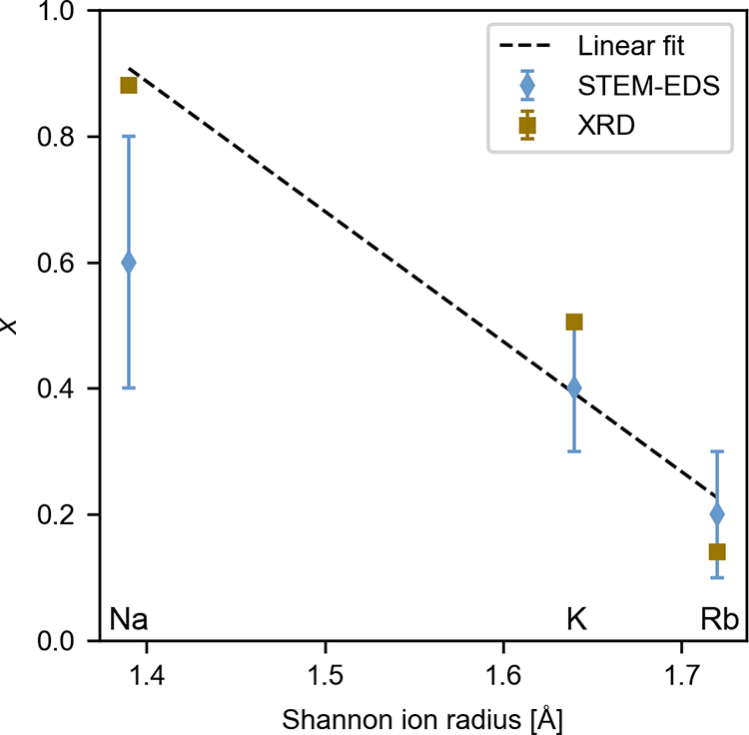
Occupancy of alkali metals on the A1 site, *x*, plotted against their respective Shannon ion radii. Data from both Rietveld refinement (XRD) and STEM–EDS are shown, where the error of the XRD data is too small for the error bar to be visible. The stippled line shows a linear fit from the XRD data with *r*^2^ = 0.92.

The concentration maps in Figure 3 are somewhat noisy, thus it was desirable to sum up all the EDS spectra at the A1 sites and the A2 sites, respectively. Atomap [14] was utilized to determine the (*x*,*y*) positions of the different sites from the HAADF–STEM images. Because the HAADF–STEM images and spectral images were acquired simultaneously and treated with the same corrections by the SmartAlign algorithm, the (*x*,*y*) positions in the HAADF–STEM images coincide with the (*x*,*y*) positions in the spectral images. Hence, the EDS spectra at the A1 sites and the A2 sites could be summed up, separately, resulting in one sum spectrum for the A1 site and one spectrum for the A2 site for each compound. The concentrations of M and Ba at the A1 and A2 sites were calculated for the three compounds, and the occupancies at each site are presented in Table 1 together with the occupancies from the Rietveld refinement. The STEM–EDS results are presented as average values which are calculated based on the concentrations measured at the A1 and A2 sites for the different compounds. The error in occupancy from EDS is defined as the difference between the measured and average values (see Supporting Information Tables S5 and S6 for details on the quantification). Based on the data presented in Table 1, the occupancy of alkali metal on the A1 site is plotted against their respective Shannon ionic radius^18^ in Figure 4, where it is demonstrated that the occupancy of the alkali metal on the A1 site decreases with increasing ionic radius of the alkali metal cation.

## Discussion

The Rietveld refinement of the X-ray diffractograms and the STEM–EDS analysis demonstrated a consistent degree of cation intermixing in the three compounds. The smaller the alkali metal is, the more likely it is to occupy the smaller A1 site (compared to the larger A2 site). The data from the STEM–EDS suffer from larger errors compared to the values obtained from Rietveld refinement, and in addition, the Na content on the A1 site is underestimated by STEM–EDS compared to XRD. There could be several reasons for this. The use of *k*-factors calculated from first principles (i.e., the *k*-factors taken directly from a software) is known to produce systematic errors up to 10–20%.^19−22^ In addition, another issue with the *k*-factor quantification procedure is that it does not account for X-ray absorption, which is particularly important for low energy X-rays, such as O_Kα_(0.525 keV) and Na_Kα_ (1.040 keV).^22^ When performing site-specific quantification, it was assumed that the sum of Ba and M corresponds to 100%. Therefore, stronger absorption of the lower energy Na_Kα_ compared to Ba_Lα_ (4.466 keV), in STEM–EDS, may have caused the underestimation of the Na concentration. A more detailed discussion of the possible sources of errors regarding the EDS quantification is presented in the Supporting Information, together with comments on the issues related to channeling effects and quantification using atomically resolved EDS data.^23,24^ As displayed in Table 1 and Figure 4, the calculated error from the Rietveld refinement is small.

The ionic radii of the A-cations in the three compounds are presented in Table 3. The radius of Ba^2+^ is significantly larger than Na^+^, smaller than Rb^+^, and close to the same size as K^+^. With increasing size follows a larger occupancy of M at the A2 sites and a simultaneous decrease of M at the A1 site, as shown in Table 1. Thus, there is an apparent relation between the cation size and the distribution of cations on the two lattice sites.

**Table 3:** Shannon Ion Radii of the Cations Present in BNN, BKN, and BRN,^18^ and the Size Difference between Ba and the Alkali Metals Δ*R*

ion	ionic radius [Å]	coordination	Δ*R* [Å]
Na^+^	1.39	12	+0.22
K^+^	1.64	12	–0.03
Rb^+^	1.72	12	–0.11
Ba^2+^	1.61	12	

Schmalzried^25^ proposed that the degree of cation disorder in the spinel crystal structure can be treated as a chemical equilibrium. Olsen et al. introduced the same concept for cation disorder in TTBs with five divalent cations and a cation vacancy.^7^ For the filled TTBs studied here, the equilibrium can be written as
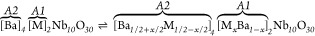
1where *x* is introduced as a distribution parameter, which ranges from 0 to 1. It is important to note that *x* does not scale linearly with disorder, it merely describes the distribution of cations between the different sites. In the case where *x* = 1, the structure is ordered, with only M occupying A1 and only Ba occupying A2, as described on the left side of (1). On the other hand, when *x* = 0, partial order is obtained because only Ba occupies A1, and equal amounts of Ba and M occupy A2,^26^ as described by the chemical formula in (2)
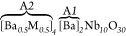
2

The Gibbs free energy change Δ*G* for the chemical equilibrium presented in (1) can be stated as
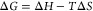
3where Δ*H* is the enthalpy change for the reaction, *T* is the temperature, and Δ*S* is the change in entropy, which is assumed to be mainly dependent on the distribution of the different cations at the different A sites (configurational entropy). This assumption has been shown to be valid for spinels,^8^ and the configurational entropy model has also been introduced for TTBs.^7^ We propose that Δ*H* depends on the relative size difference Δ*R* = *R*_Ba_ – *R*_M_ between the ionic radii of Ba and M. Considering first the situation at *T* = 0 K, the reaction is driven only by the enthalpic term, such that when Δ*R* > 0 the system is ordered (*x* = 1) and when Δ*R* < 0 the system is partially ordered (*x* = 0). If Δ*R* = 0, the system is completely disordered. At elevated temperatures, the entropy contribution must be considered. The entropy change of the cation interaction, Δ*S*_int_, in reaction 1 can be expressed as
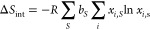
4which is the purely configurational entropy of mixing for an ideal solution, where *x*_*i*,*S*_ is the fractional occupancy of species *i* on site s, and *b*_S_ is the multiplicity of site s, leaving all other effects, such as volume and non-configurational entropy, neglected. Differentiation of the entropy (shown in eq S1 in Supporting Information) shows that the maximal configurational entropy is obtained for *x* ≈ 0.4.

The size differences Δ*R* for the materials studied here are presented in Table 3. The data presented in Table 1 demonstrate that BNN is close to being in an ordered state with *x* close to 0.9 and that BRN with *x* close to 0.1 is proximate to the partially ordered state. This is in line with our proposed relation between Δ*R* and Δ*H*, where Δ*R* is positive for BNN and negative for BRN. For BKN, *x* is close to 0.5 which, in contrast to the two other compounds, is much closer to the disordered state. This demonstrates that the dependency of the contribution of Δ*H* increases with increasing |Δ*R*|, meaning that a larger size difference between the two cations occupying the A sites drives the system toward a more ordered or partially ordered state. It is the stoichiometry of the structure that causes the system to become partially ordered in situations where M is larger than Ba. In line with the nomenclature used for spinels, we propose to call this cation order, presented in the chemical formula in (2), a perfectly inverse filled TTB.^8^

Disorder is well known in spinels, where, in spinels containing divalent and trivalent cations, there is a tendency for the larger ion to prefer the tetrahedral site, whereas the reverse holds for spinels containing divalent and four-valent cations, meaning that the oxidation state of the cations can affect the cation distribution.^8^ Crystal field stabilization energy might also determine occupation site distribution in these materials. Gardner and Morrison investigated the series Ba_4_RE_0.67_Nb_10_O_30_ (RE = La, Nd, Sm, Gd, Dy, and Y),^27^ where the most improved and stable Rietveld refinements of diffraction data were obtained when Ba was kept at the A2 site and the A1 sites were partially occupied by the RE and cation vacancies to achieve the nominal stoichiometry. This study demonstrated that the trivalent rare-earth cations occupied the smaller A1 site, due to their smaller ionic radius compared to Ba^2+^, evidencing that size difference is an important parameter determining the distribution and that it does not appear that the different cation valency affects the cation disorder in this case.

The cation occupancy observed in this study is not only determined by the thermodynamics rationalized by the ionic size. The kinetics of the cation intermixing between the A1 and A2 sites will also play a role.^7,10^Equation 3 shows that the cation disorder is temperature dependent and that the entropy term will favor disorder with increasing temperature. Equilibrium is obtained at the sintering temperature when preparing the material, but during cooling the cation distributions on the A1 and A2 sites are frozen in due to rapid slowdown of the self-diffusion of cations between the two sites. Based on the literature,^9,10^ the cation distribution we observed corresponds to an entropic state frozen in at temperatures above ∼1000 K.

The phenomenology of cation disorder in oxides has mainly been studied in detail in spinels, whereas cation disorder in TTBs has to a large degree been neglected in the literature. The systematic investigation of the site occupancy reported here has demonstrated that cation disorder also is a phenomenon taking place in TTBs. The cation disorder can be tailored by chemistry and by the thermal history of the materials.^10^ Cation disorder on A1 and A2 sites has also been shown to influence the electrical properties,^9,10^ which is interesting from an application point of view. For example, the relaxor behavior of TTBs can possibly be tailored by the cation disorder.

## Conclusions

BNN, BKN, and BRN were successfully synthesized by a two-step solid-state method. BNN and BKN were phase pure, whereas a minor secondary phase was present in BRN. A consistent distribution of cations on the A1 and A2 sites in the TTB crystal lattice was demonstrated both by Rietveld refinement and STEM–EDS, confirming that the cations are distributed such that larger cations preferentially occupy the larger A2 sites. The present findings demonstrate that the cation intermixing is an important phenomenon in TTBs. The cation order–disorder phenomenology was discussed based on a thermodynamic model developed by O’Neill and Navrotsky, originally formulated for cation interchange in spinels.

## Supporting Information Available

Details of Rietveld refinement, EDS and CL quantification, EDS spectra, further discussion on errors and inaccuracy in atomically resolved EDS, and derivation of thermodynamic equations (PDF)
