# Supplementary material for: Cation Disorder in Ferroelectric Ba4M2Nb10O30 (M = Na, K, and Rb) Tetragonal Tungsten Bronzes
Source: Inorg Chem. 2022 Sep 22;61(39):15540–6. doi: 10.1021/acs.inorgchem.2c02266 (PMC9533300; doi:10.1021/acs.inorgchem.2c02266)
Supplement: Supplementary file 1 — ic2c02266_si_001.pdf [file ic2c02266_si_001.pdf]

# Supporting information
# Cation disorder in ferroelectric $Ba_4M_2Nb_{10}O_{30}$ ($M$=Na, K, Rb) tetragonal tungsten bronzes

Inger-Emma Nylund[1†], Nora Statle Løndal[1†], Julian Walker[1], Per Erik Vullum[2,3], Mari-Ann Einarsrud[1], and Tor Grande[1*]

[1]Department of Materials Science and Engineering
[2]Department of Physics
[3]SINTEF Industry, NO-7034 Trondheim, Norway
NTNU Norwegian University of Science and Technology, NO-7491 Trondheim, Norway

## Rietveld refinement

Table S1. The details for Rietveld refinement of occupancy for the B*M*N powder samples. For the *Cmm2* structure *A2*-sites are split in two, while for the *P4bm* structure these are combined.

| Site | Atom | Occupancy |
|---|---|---|
| A2_1 | Ba | 1-(1-x)/2 |
| | *M* | (1-x)/2 |
| A2_2 | Ba | 1-(1-x)/2 |
| | *M* | (1-x)/2 |
| A1 | *M* | x |
| | Ba | 1-x |

Table S2. Refined *Cmm2* crystal structure of $Ba_4Na_2Nb_{10}O_{30}$. The number of independent parameters that were refined was 61. Unit cell parameters: a = 17.60196(19) Å, b = 17.63260(18) Å and c = 3.99081(3) Å. GOF: 2.11, Rwp: 9.69. Refined parameters are in **bold**.

| Site | Mp | Atom | X | Y | Z | Occupancy | Beq |
|---|---|---|---|---|---|---|---|
| A2_1 | 4 | Ba | **0.1731(2)** | 0.50000 | 0.50000 | **0.9398(18)** | 0.44 |
| | | Na | | | | **0.0602(18)** | 0.44 |
| A2_2 | 4 | Ba | 0.50000 | **0.6719(2)** | 0.50000 | **0.9398(18)** | 0.44 |
| | | Na | | | | **0.0602(18)** | 0.44 |
| A1 | 4 | Na | 0.75000 | 0.25000 | **0.469(7)** | **0.880(4)** | 1.6 |
| | | Ba | | | | **0.120(4)** | 1.6 |
| Nb1_1 | 2 | Nb | 0.00000 | 0.00000 | **0.985(10)** | 1 | 0.42 |
| Nb2_1 | 2 | Nb | 0.00000 | 0.50000 | **0.991(11)** | 1 | 0.42 |
| Nb2_1 | 8 | Nb | **0.89186(19)** | **0.31775(19)** | **0.990(7)** | 1 | 0.42 |
| Nb2_1 | 8 | Nb | **0.6816(2)** | **0.39212(19)** | **0.988(6)** | 1 | 0.42 |
| O1_1 | 4 | O | **0.278(2)** | 0.50000 | **0.03(4)** | 1 | 1.17 |
| O1_2 | 4 | O | 0.50000 | **0.782(2)** | **0.03(3)** | 1 | 1.17 |
| O2_1 | 8 | O | **0.8558(15)** | **0.2122(15)** | **0.048(16)** | 1 | 1.17 |
| O2_2 | 8 | O | **0.7902(17)** | **0.3576(16)** | **0.099(10)** | 1 | 1.17 |
| O3_1 | 8 | O | **0.9257(15)** | **0.0800(15)** | **-0.00(2)** | 1 | 1.17 |
| O3_2 | 8 | O | **0.9185(16)** | **0.4290(16)** | **0.048(9)** | 1 | 1.17 |
| O4_1 | 8 | O | **0.1034(12)** | **0.3002(10)** | **0.50(4)** | 1 | 1.17 |
| O4_2 | 8 | O | **0.6679(10)** | **0.6156(12)** | **0.50(4)** | 1 | 1.17 |
| O5_1 | 2 | O | 0.00000 | 0.50000 | **0.50(6)** | 1 | 1.17 |
| O5_2 | 2 | O | 0.50000 | 0.50000 | **0.52(3)** | 1 | 1.17 |

† Jointly contributed as first authors
* Author to whom correspondence should be addressed: tor.grande@ntnu.no

Table S3. Refined *P4bm* crystal structure of $Ba_4K_2Nb_{10}O_{30}$. The number of independent parameters that were refined was 32. Unit cell parameters: a = 12.54854(7) Å and c = 4.01869(3) Å. GOF: 2.26, Rwp: 9.85. Refined parameters are in **bold**.

| Site | Mp | Atom | X | Y | Z | Occupancy | Beq |
|---|---|---|---|---|---|---|---|
| A2 | 4 | Ba | **0.17208(10)** | **0.67208(10)** | **0.441(3)** | **0.7525(19)** | 0.44 |
| | | K | | | | **0.2475(19)** | 0.44 |
| A1 | 2 | K | 0.00000 | 0.00000 | **0.441(4)** | **0.505(4)** | 1.6 |
| | | Ba | | | | **0.495(4)** | 1.6 |
| Nb1 | 2 | Nb | 0.00000 | 0.50000 | **-0.035(3)** | 1 | 0.42 |
| Nb2 | 8 | Nb | **0.07497(10)** | **0.21242(10)** | **-0.046(2)** | 1 | 0.42 |
| O1 | 8 | O | 0.3406 | 0.0068 | -0.094 | 1 | 1.17 |
| O2 | 8 | O | 0.1409 | 0.0710 | -0.100 | 1 | 1.17 |
| O3 | 4 | O | 0.2856 | 0.7856 | -0.093 | 1 | 1.17 |
| O4 | 8 | O | 0.00000 | 0.50000 | 0.414 | 1 | 1.17 |
| O5 | 2 | O | 0.2933 | 0.4274 | 0.419 | 1 | 1.17 |

Table S4. Refined *P4bm* crystal structure of $Ba_4Rb_2Nb_{10}O_{30}$. The number of independent parameters that were refined was 32. Unit cell parameters: a = 12.59930(9) Å and c = 4.02539(3) Å. GOF: 2.60, Rwp: 10.80. Refined parameters are in **bold**.

| Site | Mp | Atom | X | Y | Z | Occupancy | Beq |
|---|---|---|---|---|---|---|---|
| A2 | 4 | Ba | **0.17217(10)** | **0.67217(10)** | **0.419(4)** | **0.570(4)** | 0.44 |
| | | Rb | | | | **0.430(4)** | 0.44 |
| A1 | 2 | Rb | 0.00000 | 0.00000 | **0.423(5)** | **0.141(7)** | 1.6 |
| | | Ba | | | | **0.859(7)** | 1.6 |
| Nb1 | 2 | Nb | 0.00000 | 0.50000 | **-0.056(5)** | 1 | 0.42 |
| Nb2 | 8 | Nb | **0.07484(12)** | **0.21470(11)** | **-0.068(4)** | 1 | 0.42 |
| O1 | 8 | O | 0.3406 | 0.0068 | -0.094 | 1 | 1.17 |
| O2 | 8 | O | 0.1409 | 0.0710 | -0.100 | 1 | 1.17 |
| O3 | 4 | O | 0.2856 | 0.7856 | -0.093 | 1 | 1.17 |
| O4 | 8 | O | 0.00000 | 0.50000 | 0.414 | 1 | 1.17 |
| O5 | 2 | O | 0.2933 | 0.4274 | 0.419 | 1 | 1.17 |

## EDS quantification procedure using HyperSpy and Atomap

Windows for background subtraction and integration of the peaks in the EDS data were estimated based on the total sum spectrum from each specimen, and by using the built-in functions in HyperSpy. The integration window width was set to $2FWHM$ and the background subtraction windows were positioned such that they did not include neighboring peaks. The background and integration windows used are shown in Figure S1. The quantification for each specimen was performed using the $\mathrm{Ba}_{L\alpha}$, $M_{K\alpha}$, $\mathrm{Nb}_{K\alpha}$, and $\mathrm{O}_{K\alpha}$ peaks. The main goal was to determine the chemical composition at the $A1$- and $A2$-sites, thus Atomap was used to determine the positions of the different sublattices. Since Atomap originally is written for simpler crystal structures, the function add_atoms_with_gui() was utilized in order to select atoms according to sublattice $A1$ and $A2$. In addition, masks were applied in the atom refinement procedure to remove certain sublattices, where necessary, to enable a robust fitting of selected sublattices. The atomap.tools.integrate() function was then used to sum up all spectra at each sublattice to get a less noisy and more intense spectrum to quantify the composition at each site. For all specimens it was assumed that Ba and $M$ made up 100 % of the concentration at the $A1$- and $A2$-sites. When quantification was performed pixel by pixel, the quantification algorithm sometimes failed to produce a physical concentration between 0 and 100 at%, since some of the pixels in the spectral images had very few counts. In these cases, where the pixels in the quantification maps had values <0 or >100, the pixel value was set to 0.

## EDS sum spectra for the different compositions

Figure S1 shows the EDS spectra from each compound. Also indicated are the integration windows (stippled colored lines) and background windows (full colored lines) used to determine the background (black horizontal line) at each peak used in the quantification process.

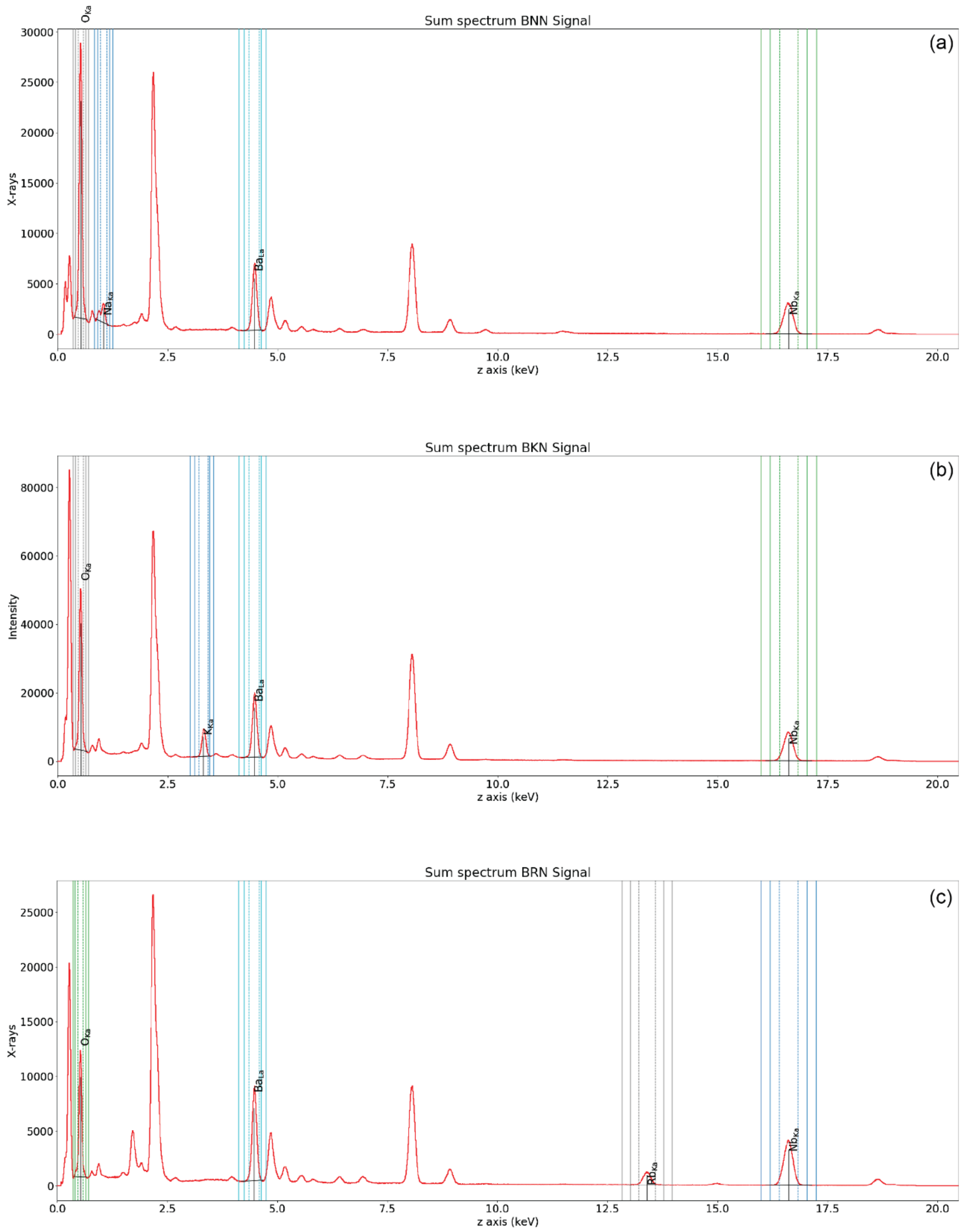


Figure S1. EDS sum spectra from (a) BNN, (b) BKN, and (c) BRN. The stippled vertical lines indicate the integration windows used and the full lines indicate the background subtraction windows. The black horizontal lines show how the background is defined based on the background windows.

## CL quantification on the A1 and A2 sites

Equation (1) in the main text provides four equations, shown in Table S5, for calculating the $x$ value in the chemical formula, for each compound, dependent on the measured concentration from the EDS data. The concentrations of the alkali metals and Ba are denoted $C_M$ and $C_{\text{Ba}}$, respectively.

Table S5. Equations for determining $x$, based on the chemical formula in Equation (1) in the main text.

| Site | Equation |
|---|---|
| $A1$ | $x = C_M$ [at%] |
| $A1$ | $1 - x = C_{\text{Ba}}$ [at%] |
| $A2$ | $\frac{1}{2} - \frac{x}{2} = C_M$ [at%] |
| $A2$ | $\frac{1}{2} + \frac{x}{2} = C_{\text{Ba}}$ [at%] |

The results from the site-specific Cliff-Lorimer quantification are presented in Table S6, where the concentrations are given as values between 0 and 1. Three decimals are kept during calculations, and later removed in the main text to reflect the precision of the quantitative EDS technique.

Table S6. Calculated concentrations for the two different sites based on the EDS measurements, and $x$ values (from equation (1) in main text) calculated from the four equations shown in Table I. $\bar{x}$ is the average of $x$ from the two different sites.

| Site | $A1$ | | | $A2$ | | | Average |
|---|---|---|---|---|---|---|---|
| | $C_M$ | $C_{\text{Ba}}$ | $x$ | $C_M$ | $C_{\text{Ba}}$ | $x$ | $\bar{x}$ |
| BNN | 0.474 | 0.526 | 0.474 | 0.100 | 0.901 | 0.801 | 0.638 |
| BKN | 0.341 | 0.659 | 0.341 | 0.242 | 0.758 | 0.515 | 0.428 |
| BRN | 0.126 | 0.875 | 0.126 | 0.355 | 0.645 | 0.289 | 0.207 |

## Discussion of possible errors and sources of inaccuracy regarding atomically resolved EDS quantification

The STEM-EDS measurements suffers from larger errors than the XRD analysis and in addition the Na content on the $A1$-site is underestimated by STEM-EDS compared to XRD. There could be many reasons for this. A simple linear $k$-factor approach [1] was used to determine the concentrations of the different elements on the different sites. No assessment of the $k$-factors was performed, however, values calculated from first principles are known to produce systematic errors up to 10-20 % [2–5]. Experimentally determined $k$-factors can result in relative errors as small as 1 % [5–7], however, this is a difficult and time-consuming procedure as multicomponent standard specimens with known compositions must be used, and thus, experimental $k$-factors were not obtained here. Another issue with the $k$-factor quantification procedure is that it does not account for X-ray absorption, which is particularly important for low energy X-rays, such as $\text{O}_{K\alpha}$(0.525 keV) and $\text{Na}_{K\alpha}$ (1.040 keV) [5]. Surprisingly, in Table 2 in the main text, the average O composition in BNN is overestimated while the Na concentration is underestimated, even though the energy of $\text{O}_{K\alpha}$ is lower than that of $\text{Na}_{K\alpha}$. This error is suggested to be a result of errors in the $k$-factor. Furthermore, since alkali metals are known to be volatile, and the specimens were irradiated by the electron beam for an extensive period of time, 9 spectral images acquired early during the experiment and 9 spectral images acquired late were summed up, respectively, for all the compounds, and the average compositions were calculated. For BNN, this procedure demonstrated an initial concentration of Na of 3.0 at%, and later the composition was reduced to 2.4 at%. For BKN and BRN, the respective concentrations of K and Rb were initially 4.1 at% and 5.3 at%, which in both cases increased to 4.2 at% and 5.8 at%, respectively. This suggests that Na is more volatile than K and Rb during the electron beam irradiation and may explain some of the underestimation of Na by STEM-EDS compared to XRD. The reason why the calculated concentrations

of K and Rb increased may be explained by a larger carbon contamination build up on BKN and BRN compared to BNN, as demonstrated by the large $\mathrm{C}_{K\alpha}$ peak at 0.277 keV in Figure S1(b, c) compared to the relatively weak $\mathrm{C}_{K\alpha}$ peak in Figure S1(a). This likely leads to a greater reabsorption of O during the experiment, thus a small increase in K and Rb is calculated because of the decrease in the intensity of $\mathrm{O}_{K\alpha}$. The carbon contamination may also explain why O is underestimated in BKN and BRN in the results presented in Table 2, in the main text. In addition, a higher average atomic number also leads to a higher absorption of low energy photons since the measured O concentration is the lowest in BRN, which has the highest average atomic number. When it comes to the site-specific quantification it was assumed that $Ba + M = 100\%$, therefore stronger absorption of the lower energy $\mathrm{Na}_{K\alpha}$ compared to $\mathrm{Ba}_{L\alpha}$ (4.466 keV), in addition to the error in the $k$-factor, may have caused the underestimation of Na compared to the value measured from XRD.

Another source of error in atomically resolved STEM-EDS is caused by channeling along the atomic columns when the specimen is aligned along a low-order zone axis [8,9]. Channeling is beneficial in the sense that it keeps the electron probe localized, thus atomically resolved STEM and EDS is obtainable. Additionally, channeling causes a fractional increase in the number of X-rays generated from each atom column, and since counts are often a limiting factor in EDS analysis, an increased number of photons is desirable. However, the main issue is that this increase is non-linear, thus simple ratio methods such as the $k$-factors approach no longer work well, and direct comparison with simulation becomes necessary [9]. The reason why the $k$-factor method is still applied here is due to its simple implementation and the lack of other viable solutions. Through simulations of very thin samples << 10 nm, MacArthur *et al.* [9] show that, due to channeling effects, configuration, i.e., in what order that atoms are arranged parallel to the electron beam, also plays a role on the measured intensity of a given element, thus possible ambiguity is caused if the configuration is not known, even when comparing with simulations. Channeling can be greatly suppressed by tilting the specimen about 2° away from zone axis, at the cost of a loss in resolution. Atomic resolution was demonstrated to remain in a specimen of 42 Å thickness [8], but the ceramic particles studied here were much thicker (> 500 Å), hence tilting was not applied in order to maintain atomic resolution. Interestingly, the TTBs studied here exhibit two different atom columns, $A1$ and $A2$, containing the same elements within each compound, but with different concentrations at the different sites. Looking at the measured concentrations at each site in each compound (Table S6), and the calculated value of $x$ in Equation 1 ($x$ = concentration of alkali metal on $A1$-site), may give some indications of how channeling affects the calculated intensities for each compound. Taking the results from the Rietveld refinement as a reference, Na is underestimated in the measurements at both $A1$ and $A2$. This is believed to be due to absorption, and this makes it difficult to estimate the effects of channeling. For measurements of K and Rb concentrations, the calculated value of $x$ from the $A1$-site is underestimated by 28 and 10% in BKN and BRN, respectively. The calculated $x$ value based on measurements from the $A2$-site results in an overestimation of 8 and 105%, respectively, for BKN and BRN. The TTB structure contains two $A1$-sites and four $A2$-sites, whereas the composition is four Ba and two alkali metals per unit cell. The greatest intermixing of cations (in terms of concentration closest to 50/50 of each element) therefore happens at the $A1$-site in BKN, but at the $A2$-site in BRN (see Table 1 in main text). The $x$-value calculated from the STEM-EDS data for BKN and BRN both have the largest error from the column where the intermixing is closest to 50/50, i.e., the $A1$-site in BKN, and the $A2$-site in BRN. This is consistent with results obtained for a PtNi nanoparticle, demonstrating that the error gets larger the more impure the columns are [8]. Since the data shown here were acquired on-axis, and it is known that channeling effects causes a non-linear relation between the concentration of each element in the columns and the measured intensity, comparison to simulations should be performed in order to improve the results of the quantification. All the mentioned shortcomings are recommended for future work. However, the filled TTBs are suggested as excellent candidates for a more thorough EDS study. They are assumed to be good candidates since they can be synthesized with different compositions and contain both light elements like O and Na, and heavy elements like Rb and Ba. The distance between the $A1$ and the $A2$ column is fairly large (>4 Å), making

the atom columns relatively easy to resolve, and in addition, both the $A1$- and the $A2$-sites have $BO_6$ octahedra as their closest neighbors, hence, the effect of neighboring columns should be similar for both sites and only minimally affected by probe tails and beam spreading to the opposite $A$-site, given that the specimen is sufficiently thin[9].

## Derivation of thermodynamic equations

For the chemical equilibrium in equation (1) in the main text, we write out the entropy part as follows:

$$\Delta S_{int} = -R\sum_{s} b_s \sum_{i} x_{i,s} ln x_{i,s} = -R\left\{2\left[\frac{x}{2}ln\frac{x}{2}+\frac{1-x}{2}ln\frac{1-x}{2}\right]+4\left[\frac{1+x}{8}ln\frac{1+x}{8}+\frac{1-x}{8}ln\frac{1-x}{8}\right]\right\} \quad (S1)$$

where $x$ is the degree of cation interchange, $b_s$ is the multiplicity of site (or sublattice) $s$, and $x_{i,s}$ is the fractional occupancy of species $i$ (cations or vacancies) on site $s$.

Differentiation of the equation (S1) gives $\frac{\partial \Delta S_{int}}{\partial x} = -R\left\{\frac{1}{2}ln\frac{x^2(1+x)}{(1-x)^3}\right\}$

giving that the maximal configurational entropy (at $\frac{\partial \Delta S_{int}}{\partial x} = 0$) is obtained for $x = 0.396$.

## References


(1) Cliff, G.; Lorimer, G. W. The Quantitative Analysis of Thin Specimens. *J. Microsc.* **1975**, *103* (2), 203–207.

(2) Maher, D. M.; Joy, D. C.; Ellington, M. B.; Zaluzec, N. J.; Mochel, P. E. Relative Accuracy of K-Factor Calculations for Thin-Film X-Ray Analysis. *Anal. Electron Microsc.* **1981**, 33–38.

(3) Newbury, D. E.; Williams, D. B.; Goldstein, J. I.; Fiori, C. E. Observation on the Calculation of KAB Factors for Analytical Electron Microscopy. *Anal Electron Microsc* **1984**, *2*, 276–278.

(4) MacArthur, K. E.; Slater, T. J. A.; Haigh, S. J.; Ozkaya, D.; Nellist, P. D.; Lozano-Perez, S. Quantitative Energy-Dispersive X-Ray Analysis of Catalyst Nanoparticles Using a Partial Cross Section Approach. *Microsc. Microanal.* **2016**, *22* (1), 71–81.

(5) Watanabe, M; Williams, D. B. The Quantitative Analysis of Thin Specimens: A Review of Progress from the Cliff-Lorimer to the New ζ-Factor Methods. *J. Microsc.* **2006**, *221* (2), 89–109.

(6) Wood, J. E.; Williams, D. B.; Goldstein, J. I. Experimental and Theoretical Determination of KAFe Factors for Quantitative X-Ray Microanalysis in the Analytical Electron Microscope. *J. Microsc.* **1984**, *133* (3), 255–274.

(7) Sheridan, P. J. Determination of Experimental and Theoretical KASi Factors for a 200-KV Analytical Electron Microscope. *J. Electron Microsc. Tech.* **1989**, *11* (1), 41–61.

(8) MacArthur, K. E.; Brown, H. G.; Findlay, S. D.; Allen, L. J. Probing the Effect of Electron Channelling on Atomic Resolution Energy Dispersive X-Ray Quantification. *Ultramicroscopy* **2017**, *182*, 264–275.

(9) MacArthur, K. E.; Yankovich, A. B.; Béché, A.; Luysberg, M.; Brown, H. G.; Findlay, S. D.; Heggen, M.; Allen, L. J. Optimizing Experimental Conditions for Accurate Quantitative Energy-Dispersive X-Ray Analysis of Interfaces at the Atomic Scale. *Microsc. Microanal.* **2021**, *27* (3), 528–542.
